# Immigrant status and increased risk of heart failure: the role of hypertension and life-style risk factors

**DOI:** 10.1186/1471-2261-12-20

**Published:** 2012-03-26

**Authors:** Yan Borné, Gunnar Engström, Birgitta Essén, Bo Hedblad

**Affiliations:** 1 Department of Clinical Sciences, Cardiovascular Epidemiology, Skåne University Hospital, Lund University, 20502 Malmö, Sweden; 2 Department of Women's and Children's Health, International Maternal and Child Health (IMCH), Uppsala University, 751 85 Uppsala, Sweden

**Keywords:** Immigrant status, heart failure, risk factors, cohort study, case-fatality, epidemiology

## Abstract

**Background:**

Studies from Sweden have reported association between immigrant status and incidence of cardiovascular diseases. The nature of this relationship is unclear. We investigated the relationship between immigrant status and risk of heart failure (HF) hospitalization in a population-based cohort, and to what extent this is mediated by hypertension and life-style risk factors. We also explored whether immigrant status was related to case-fatality after HF.

**Methods:**

26,559 subjects without history of myocardial infarction (MI), stroke or HF from the community-based Malmö Diet and Cancer (MDC) cohort underwent a baseline examination during 1991-1996. Incidence of HF hospitalizations was monitored during a mean follow-up of 15 years.

**Results:**

3,129 (11.8%) subjects were born outside Sweden. During follow-up, 764 subjects were hospitalized with HF as primary diagnosis, of whom 166 had an MI before or concurrent with the HF. After adjustment for potential confounding factors, the hazard ratios (HR) for foreign-born were 1.37 (95% CI: 1.08-1.73, *p *= 0.009) compared to native Swedes, for HF without previous MI. The results were similar in a secondary analysis without censoring at incident MI. There was a significant interaction (*p *< 0.001) between immigrant status and waist circumference (WC), and the increased HF risk was limited to immigrants with high WC. Although not significant foreign-born tended to have lower one-month and one-year mortality after HF.

**Conclusions:**

Immigrant status was associated with long-term risk of HF hospitalization, independently of hypertension and several life-style risk factors. A significant interaction between WC and immigrant status on incident HF was observed.

## Background

Heart failure (HF) is one of the leading causes for morbidity and mortality, particularly in the elderly. Hypertension and myocardial infarction (MI) are the main causes of HF in the general population [1-5]. Other important risk factors that have been associated with incidence of HF include age, male sex, overweight, diabetes, smoking, physical inactivity, alcohol consumption, inflammatory and socioeconomic factors [2,6-13].

It has repeatedly been shown that immigrants in Sweden have higher risk of coronary heart disease and stroke compared to Swedish-born subjects [14-17]. In a previous study of the entire population of Malmö, Sweden, we found substantial differences in risk of HF hospitalization among foreign-born subjects [18]. In that study, increased incidence of HF hospitalizations was found in immigrants from Finland, Former Yugoslavia and Hungary. However, it is still unclear to what extent the increased risk in these groups could be explained by major biological and lifestyle risk factors for HF, e.g., hypertension, overweight, and smoking.

Thus, the purpose of the present study was to further explore the association of immigration status and risk of HF hospitalization in an urban population-based cohort and to what extent the relationship is explained by conventional cardiovascular risk factors. We also explored whether immigrant status was related to case-fatality (e.g. 1-month and 1-year, respectively) after HF.

## Methods

### Study population

The Malmö Diet and Cancer (MDC) cohort is a prospective cohort study from the city of Malmö in southern Sweden. Sample characteristics, data collection and clinical definitions for MDC have been described previously [19-21]. Briefly, 28,449 men (n = 11,246, born 1923-1945) and women (n = 17,203, born 1923-1950) attended a baseline examination between March 1991 and September 1996. Participants underwent sampling of peripheral venous blood, measurement of blood pressure and anthropometric measures and filled out a self-administered questionnaire.

Subjects with history of cardiovascular events (coronary events or stroke, n = 970 subjects) or HF (n = 46 subjects) at the baseline examination were excluded. In addition, subjects were also excluded due to missing information on blood pressure (BP), waist circumference (WC), smoking habits, alcohol consumption, physical activity, leukocyte counts, educational level, marital status and country of birth. Thus, the final study population in the analysis consisted of 26,559 (10,227, 38.5% men and 16,332, 61.5% women) subjects, aged 45-73 years. The study was approved by the ethical committee at Lund University Lund, Sweden, and all participants provided informed consent.

### Measurements and definitions

Information on current use of BP lowering, lipid-lowering or anti-diabetic medications, smoking habits, alcohol consumption, leisure time physical activity, educational level, marital status and country of birth were obtained from a self-administered questionnaire [20]. WC (in cm) was measured midway between the lowest rib margin and iliac crest in the standing position without clothing. WC was stratified into normal WC and high WC (≥ 94 cm for men and ≥ 80 cm for women) [22]. Blood pressure was measured using a mercury-column sphygmomanometer after 10 minutes of rest in the supine position. Hypertension was defined as blood pressure equal or above 140/90 mm Hg or current use of blood pressure-lowering medication. Leukocyte concentrations were analysed consecutively in fresh heparinized blood. Diabetes mellitus was defined as fasting whole blood glucose level greater than 109 mg/dL (e.g. 6.0 mmol/L), self-reported physician's diagnosis of diabetes, or use of antidiabetic medications. Subjects were categorized into current smokers (i.e., those who smoked regularly or occasionally) or non-smokers (i.e., former smokers and never smokers). High alcohol consumption was defined as > 40 gram alcohol per day for men and > 30 g/day for women. Leisure time physical activity was grouped as lowest quartile or other. As previously described educational level was defined as low education (up to grade 9) and high (> 9 years) [23]. Marital status was categorized into married or unmarried. Immigrant status was grouped as Swedish-born and foreign- born. We were unable to study immigrants from individual countries of birth due to limited numbers of HF cases.

### Ascertainment of cardiovascular events and HF

The Swedish Hospital Discharge Register (SHDR) was used for case retrieval. Validation study has shown that a primary diagnosis of HF in the SHDR has a validity of 95% [24]. The corresponding figure for MI is 94% [25]. HF was defined as International Classification of Diseases- 8^th ^revision (ICD-8) code 427.00, 427.10 and 428.99; 428 (ICD-9); and I50, I11 (ICD-10) as the primary diagnosis [24]. Non-fatal MI was defined as 410 (ICD-8 and 9) or I21 (ICD-10) [25]. Information on mortality was obtained through the Swedish Cause of Death Register. All subjects were followed from the baseline examination until a first diagnosis of HF, emigration from Sweden, death or December 31^st^, 2008, whichever came first.

### Statistical analysis

Cox proportional hazards regression was used to examine the association between selected immigrant status and risk of HF hospitalization in the MDC cohort. Hazard ratios (HR), with 95% confidence interval (CI) were calculated. Age and sex were included as covariates in the basic model. Secondly, we also adjusted for systolic BP, use of BP-lowering medication, lipid-lowering medication, diabetes mellitus, WC, current smoking, high alcohol consumption, low physical activity and leukocyte counts. Possible interactions between immigrant status and age, sex and cardiovascular risk factors on incidence of HF were explored by introducing interaction terms in the multivariate model. The primary analysis was performed with censoring at first nonfatal MI during follow-up, i.e., cases with MI prior to HF were not counted. Secondary analysis included all HF incident cases, regardless of MI. Two-sided p values < 0.05 were considered significant. The Kaplan-Meier curve was used to illustrate incidence of hospitalization due to HF in relation to immigrant status and waist circumference.

Case-fatality rates were calculated as the proportion of those with a HF hospitalization that died within 1-month and 1-year, respectively. Cox proportional hazards regression was used and adjusted for age, sex and year of HF event. All analyses were performed using PASW version 18 (SPSS Inc., Chicago, Illinois).

## Results

Overall, mean age (± standard deviation) at baseline was 58 ± 7.6 years and 61.5% were women. A total of 23,430 subjects were born in Sweden and 3,129 (11.8%) were born outside Sweden. Of those born outside Sweden, the majority came from Denmark (10.5%), Former Yugoslavia (8.3%), Finland (7.6%), Germany (8.8%), Poland (5.0%) and Hungary (4.3%). Baseline characteristics of Swedish- born and foreign- born in relation to conventional cardiovascular risk factors (WC, leukocyte count, systolic BP, use of BP-lowering and lipid-lowering medication, diabetes mellitus, current smoking, high alcohol consumption, low physical activity) and socioeconomic factors (educational level, marital status) are presented in Table 1. Foreign-born subjects were younger, more often current smokers, diabetics, high alcohol consumers, and had more often low physical activity than those born in Sweden. During a mean follow-up of 15 years, a total of 764 individuals (325 men and 273 women) were hospitalized with HF as primary diagnosis. Of them, 166 (96 men and 70 women) had an incident MI before or concurrent with HF hospitalization during follow-up. The latter group was censored at the time of the infarction in the primary analysis.

**Table 1:** Characteristics of subjects in the Malmö diet and cancer (MDC) cohort in relation to immigration status, at the baseline examination 1991-1996

MDC (N = 26,559)	Swedish-born (n = 23,430)	Foreign-born (n = 3,129)	*P *value
Mean age (years)	58.2 ± 7.6	56.9 ± 7.2	< 0.001

Men (%)	38.4	39.1	< 0.001

Waist circumference (cm)	84 ± 15	85 ± 10	< 0.001

SBP (mmHg)	141 ± 20	140 ± 20	< 0.001

DBP (mmHg)	86 ± 10	85 ± 13	0.426

Leukocytes (10*^9 ^*/L)	6.4 ± 2.2	6.5 ± 3.5	0.183

Hypertension (%)	40.5	38.6	< 0.001

Use of BP-lowering medications (%)*	41.1	40.1	< 0.001

Use of lipid-lower medications (%)	2.4	2.1	0.279

Diabetes (%)	2.8	3.3	< 0.001

Current smoker (%)	27.9	31.0	< 0.001

High alcohol consumption (%)	4.2	5.2	0.015

Low physical activity (%)	24.5	28.4	< 0.001

Low educational level (%)	42.1	35.3	< 0.001

Married (%)	65.7	62.3	< 0.001

All other values are mean ± SD, unless otherwise stated. * Use of blood pressure (BP)-lowering medications is calculated as proportions of hypertensives in each group (n = 9488 and n = 1207, respectively).

### Risk of HF hospitalizations in relation to immigrant status

The overall analysis showed higher risk of HF hospitalization for foreign-born compared to Swedish- born. Adjusted for age and sex, foreign- born had a significantly higher risk for HF (HR: 1.44; 95% CI, 1.14-1.82) compared to Swedish-born. This increased risk remained (HR: 1.37; 1.08-1.73) after adjustment for other possible confounders, Table 2. If cases with MI before or concurrent with HF hospitalization (n = 166) were included in the analysis, the risk for HF hospitalization among foreign-born (HR: 1.24; 1.01-1.54) was only marginally changed, Table 2.

**Table 2:** Final multivariate model for first hospitalization due to heart failure in the MDC cohort

	INCIDENT HF WITHOUT PRIOR MI HR† (95% Cl)	*p *value	ALL INCIDENT HF HR† (95% Cl)	*p *value
Foreign-born *(yes vs no)*	1.37 (1.08-1.73)	0.009	1.24 (1.01-1.54)	0.045

Age *(per 1 year)*	1.11 (1.09-1.12)	< 0.001	1.11 (1.09-1.12)	< 0.001

Male sex *(yes vs no)*	1.71 (1.44-2.03)	< 0.001	1.68 (1.45-1.95)	< 0.001

Waist circumference *(per 5 cm)*	1.03 (1.02-1.04)	< 0.001	1.03 (1.02-1.04)	< 0.001

Systolic blood pressure *(per 10 mm Hg)*	1.13 (1.09-1.18)	< 0.001	1.15 (1.11-1.20)	< 0.001

Leukocyte count *(per 10^9 ^/L)*	1.02 (1.01-1.03)	0.005	1.02 (1.01-1.03)	0.001

Use of BP-lowering medications *(yes vs no)*	2.02 (1.69-2.41)	< 0.001	2.03 (1.74-2.37)	< 0.001

Use of lipid-lowering medications *(yes vs no)*	1.10 (0.73-1.63)	0.658	1.43 (1.06-1.94)	0.021

Diabetes mellitus *(yes vs no)*	2.78 (2.12-3.65)	< 0.001	2.80 (2.22-3.54)	< 0.001

Smoking *(yes vs no)*	1.94 (1.63-2.32)	< 0.001	2.11 (1.81-2.46)	< 0.001

High alcohol consumption *(yes vs no)*	1.53 (1.10-2.14)	0.012	1.40 (1.03-1.91)	0.032

Low physical activity *(yes vs no)*	1.27 (1.07-1.52)	0.008	1.26 (1.07-1.47)	0.004

Unmarried *(yes vs no)*	1.21 (1.02-1.44)	0.028	1.15 (0.98-1.34)	0.081

Low educational level *(yes vs no)*	1.18 (1.00-1.39)	0.050	1.23 (1.06-1.42)	0.005

Hazard ratio (HR)† in the final model. Cl, confidence interval.

In the final model, age and male sex, increased WC, leukocyte count, systolic BP, use of BP-lowering medication, diabetes, smoking, high alcohol consumption, low physical activity, low educational level were independently associated with an increased risk for HF, Table 2.

### Interaction between immigrant status and other risk factors on incidence of HF

Interaction terms between covariates were added in the final Cox's proportional hazards model with adjustment for possible confounders. There was a statistically significant interaction between immigrant status and WC (*p *< 0.001) on incidence of HF. There were no other significant interactions between immigrant status and risk factors.

**Figure 1:**
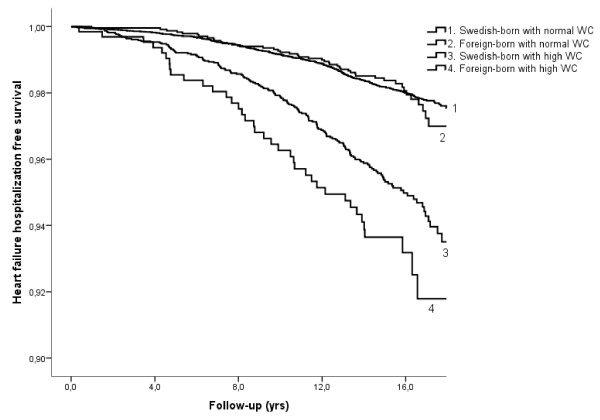
**Heart failure hospitalization free survival in relation to immigration status and high/normal waist circumference**.

To further explore the interaction between country of birth and WC, WC was stratified into normal and high WC in men and women, respectively [22], Table 3 and Figure 1. After stratification for WC, a significant higher risk of HF was only observed in foreign-born with high WC (HR: 2.11; 95% CI, 1.62-2.76), while foreign-born with normal WC had similar risk (HR: 1.17; 0.85-1.60) as compared to Swedish natives with normal WC.

**Table 3:** Interaction between immigration status and waist circumference (WC) on incidence of HF in the MDC cohort

	INCIDENT HF WITHOUT PRIOR MI HR† (95% Cl)	ALL INCIDENT HF HR† (95% Cl)
Interaction term Immigrant status*WC	*P *< 0.001	*P *< 0.001

Swedish-born with normal WC (reference)	1	1

Swedish- born with high WC	1.67 (1.38-2.02)	1.71 (1.45-2.03)

Foreign- born with normal WC	1.17 (0.85-1.60)	1.06 (0.79-1.42)

Foreign- born with high WC	2.62 (1.87-3.67)	2.45 (1.80-3.34)

Hazard ratio HR† adjusted for age, sex, civil status, education level, smoking habits, alcohol consumption, physical activities, BP-lowering medication, lipid-lowering medication, systolic BP, leukocyte count and diabetes mellitus. Cl, confidence interval.

### Case fatality

Thirty-two (4.2%) subjects died within 1-month after the HF hospitalization and 95 (18.9%) had died one year after the HF. After adjustment for age, sex and year of the HF hospitalization, the immigrants group tended to have lower one-month and one-year mortality (HR: 0.20; 95% CI: 0.03-1.44, *p *= 0.109 and HR: 0.47; 0.22-1.01, *p *= 0.053, respectively).

## Discussion

The present population-based cohort study shows that being foreign-born is associated with significantly higher risk for HF hospitalization, independent of several biological, lifestyle and socioeconomic risk factors. The results are in line with prior studies on immigration status and cardiovascular disease (CVD) in Sweden [14,16,18]. However, the present results also show that the increased risk among immigrants is modified by the presence of other risk factors. There was a significant interaction between WC and immigrant status on risk of HF hospitalizations, and the increased incidence was mainly observed in those with high WC.

One possible explanation for the increased risk of HF hospitalization in foreign-born compared to Swedish-born might be influences from their country of birth. Compared to 15.6% being foreign-born in whole Malmö [18], the proportion of foreign-born in the MDC cohort were 11.8% of all study subjects. This group mainly came from Denmark, Former Yugoslavia, Finland, Germany, Poland and Hungary. The majority of these countries have higher incidence of CVD compared to Sweden [26,27]. Since most cases of HF are caused by hypertension or CHD, the high CVD risk in their country of origin might partly explain the increased risk of hospitalization due to HF. It has often been suggested that socioeconomic differences could explain the high morbidity in immigrant groups. Studies have shown that residential areas in Malmö with high proportion of immigrants and low socioeconomic status have high incidence of CVD [28,29]. However, the immigrants in this cohort study had higher education levels than those born in Sweden and the present results remained significant also after adjustments for education and marital status. Socioeconomic differences therefore seem to be an insufficient explanation for the increased incidence of HF hospitalizations in foreign-born.

In the present study, a wide range of biological and life-style risk factors were independently associated with risk for HF. The increased HF risk for foreign-born still remained after adjustment for these risk factors. There was a significant interaction between immigrant status and WC on incidence of HF, which showed that the highest risk for HF was limited to foreign-born with high WC. As a heterogeneous group there are substantial differences among immigrants to Sweden by country of origin [30,31]. A previous cross-sectional study, based on the MDC cohort, found that women born in Hungary, Poland and Germany had higher WHR compared to Swedish-born women, after taking age, height, smoking, physical activity, occupation and percentage of body fat into account [31]. In men, WHR was increased in participants from Yugoslavia, Germany and Finland [31]. In that study length of residence in Sweden was found inversely associated with central adiposity in immigrants and it was concluded that immigrants may be at higher risk of obesity-related comorbidities [31].

Several studies have shown that increased abdominal adiposity is strongly associated with cardiovascular risks [10,32,33]. Inadequate exercise, over-intake of food or alcohol, metabolic imbalance and genetic abnormalities could cause high WC. The high WC influence known risk factors, e.g., dyslipidemia, hypertension, glucose intolerance, inflammation markers [13,34,35], that increase risk of developing HF.

Foreign-born tended to have lower mortality after HF compared to Swedish- born, but the difference did not reach statistical significance. This might be explained by the so-called "obesity paradox", since the foreign-born had higher WC than Swedish-born and overweight and high WC paradoxically have been associated with improved outcome among HF patients [36,37]. It has been reported that immigrants and native Swedish HF patients are quite similar in terms of symptoms, health care seeking, the distress level, physical function, emotional state and self care [38,39]. More immigrants than Swedes are referred to HF clinic after discharge for follow-ups [40], which could reduce mortality in this group.

### Strength and limitation

The study used large numbers of subjects with a long follow-up period and identified large numbers of HF events [19,21]. The cardiovascular endpoints were retrieved from national registers, and studies have showed high case validity for HF and MI in the register data [24,25].

A main limitation of the present study is lack of information on type and cause of HF. Previous studies have demonstrated that immigrants to Sweden have an increased incidence of CVD [16,17]. However, we can only speculate whether the increased risk of hospitalizations due to HF among immigrants in the present study was related to a reduced or normal ejection fraction. In addition, we were unable to include HF patients who only were treated as out-patients. The total incidence of HF is therefore underestimated and we cannot make any conclusion about less severe cases which often are treated as out-patients. The 40.8% participation rate in the MDC study questions the representativity of the population [41]. It was shown that non-participants had higher mortality rate than participants in the MDC cohort. However, there was no substantial difference when comparing baseline characteristics of subjects in the MDC study to a survey study from the Malmö city with participation rate of 75% [41]. Another short-coming is that we were unable to study immigrants by country of origin due to limited number of HF events, however in a previous study based on the whole Malmö city population we found an increased incidence of HF hospitalizations in immigrants from Finland, Former Yugoslavia and Hungary [18].

The MDC study required participants to be able to speak Swedish language. One question is whether this group of immigrants is representative to all immigrants in the city. Among all subjects aged 45-73 years in the whole Malmö population, foreign- born had a significantly higher risk for HF (HR: 1.27; 95% CL, 1.17-1.38) compared to Swedish-born after adjustment for age and sex. The corresponding HR in the MDC cohort was 1.44 (95% CI; 1.14-1.82), and we therefore believe that the results can be generalized.

The choice of risk factors variables in the multivariate model can influence the results since adjustments for risk factors that are mediators in the causal pathway will underestimate of the relation, while leaving out genuine confounders will overestimate the result. The variables used for adjustments in the study, e.g., age, sex, smoking, hypertension, diabetes, abdominal obesity, alcohol consumption and physical activity are well known cardiovascular risk factors [2,4,5,9,10,12,42,43]. Educational level is a widely used measure of socioeconomic circumstances in epidemiologic studies, and is considered to be related to health outcome by its influence on lifestyle behaviors and value [44]. Low educational level has been reported to associate with higher cardiovascular risk [45,46]. Marital status has been found associated with HF [7,47].

The lack of follow-up data regarding anthropometric measures and other risk factors in the present study is another issue to be discussed. It is possible that biological factors, e.g., blood pressure and WC changed during the follow-up. However, this is usually a slow process and one study found that adipose tissue distribution is stable through the lifespan [48]. Some subjects might change the status in terms of smoking, physical activity, alcohol consumption and marriage. It is unknown whether change of risk factors during the follow-up could be differential between immigrants and native Swedes.

## Conclusions

In conclusion, immigrant status is associated with long-term risk of HF hospitalization, independently of hypertension and several life-style risk factors. A significant interaction between WC and immigrant status on incident HF was observed.

## Competing interests

Gunnar Engström is employed as senior epidemiologist by AstraZeneca R&D.

## Authors' contributions

YB, GE and BH constructed the concept and design of the project; YB performed the analysis and drafted the manuscript; YB, GE, BE and BH participated in the analysis and interpretation of data and revised the manuscript critically. All authors approved the final manuscript to be published.

## Pre-publication history

The pre-publication history for this paper can be accessed here:

http://www.biomedcentral.com/1471-2261/12/20/prepub
